# ‘A virtue beyond all medicine’: The Hanged Man's Hand, Gallows Tradition and Healing in Eighteenth- and Nineteenth-century England

**DOI:** 10.1093/shm/hkv044

**Published:** 2015-05-02

**Authors:** Owen Davies, Francesca Matteoni

**Keywords:** corpse medicine, healing touch, gallows, executioners, wens

## Abstract

From the eighteenth century through to the abolition of public executions in England in 1868, the touch of a freshly hanged man's hand was sought after to cure a variety of swellings, wens in particular. While the healing properties of corpse hands in general were acknowledged and experimented with in early modern medicine, the gallows cure achieved prominence during the second half of the eighteenth century. What was it about the hanged man's hand (and it always was a male appendage) that gave it such potency? While frequently denounced as a disgusting ‘superstition’ in the press, this popular medical practice was inadvertently legitimised and institutionalised by the authorities through changes in execution procedure.

*James White*, aged 23, and *Walter White*, his brother, aged 21, were executed at *Kennington Common*, for breaking open and robbing the dwelling house of farmer *Vincent* of *Crawley*. They acknowledged the justice of their sentence, but laid their ruin to an accomplice, who, they declared, decoyed them from their labouring work, by telling them how easily money was to be got by thieving.—While the unhappy wretches were hanging, a child about nine months old was put into the hands of the executioner, who nine times, with one of the hands of each of the dead bodies, stroked the child over the face. It seems the child had a wen on one of its cheeks, and that superstitious notion, which has long prevailed, of being touched as before mentioned, is looked on as a cure.

*Gentleman's Magazine*, 19 April 1758

The recent digitisation of millions of pages of eighteenth- and nineteenth-century British newspapers and periodicals opens up new avenues for tracing popular medical practices that were formerly, and largely, only understood by historians through the lens of printed antiquarian and folklore collections.^1^ Systematic and painstaking research through local newspapers before the advent of digitisation had revealed the wealth of material to be discovered regarding ‘folk’ or ‘popular’ medical practices and practitioners on a local or county basis.^2^ But the digitisation of the provincial press enables, for the first time, the confident mapping of national and regional practices through analysing reportage, editorials and advertisements. These reflect immediate, contemporary beliefs and actions, as compared with the significant chronological distance and second-hand distortions that sometimes separate folklore reports and the acts and beliefs recorded.

A thorough search of the digitised newspaper and periodical archives reveals 27 instances of stroking, such as that cited above, being carried out or requested at public hangings in order to cure swellings, goiters (bronchocele), scrofula, skin tumours and other excrescences—wens (sebaceous cysts on the scalp or face) in particular. The first recorded case was in 1758 and the last in 1863. The medical market for curing such afflictions was considerable, provoking John Morley to publish his *Essay on the Nature and Cure of Scrophulous Disorders, Vulgarly called the King's Evil* in 1770. His stated aim was to ensure ‘the poor Labourers and Handicraftsmen might not throw away their Money and Time after a parcel of specious Advertisements, calculated to pick the poor Patients Pockets’. He had in mind the likes of Henry Season the Devizes ‘Physician and Student in the Astreal Sciences’, who advertised in his almanac for 1762 that ‘He cures Wens as formerly, and Scrophulous Swellings; and has cured more without cutting than any one in *Wilts*.’^3^ For sufferers of facial wens and the like, particularly women and children, there was considerable concern about the irrevocable scarring that either the untreated affliction or surgery would cause.^4^ A century after Season's medical days, the cure of large wens was still very much a choice between the scalpel and the slow and relatively expensive application of caustics and plasters that required between 30 to 50 days of regular treatment.^5^ Considering this, it is understandable that many sufferers would steel their nerves and seek out the hanged man's hand.

While the recorded instances of the hanged man's stroke represent only a tiny fraction of the thousands of executions over this period, it is likely that the practice was far more common than the newspaper record suggests. The folklore and antiquarian archives confirm that both the notion and the practice were widely known in English popular cultures. Writing in the mid-nineteenth century the Northamptonshire folklorist Thomas Sternberg stated, for example, that he knew of ‘many persons’ who had received the cure successfully. The novelist Thomas Hardy also indicated the frequency of the cure in his short story the ‘Withered Arm’ where the central character Gertrude is advised by the cunning-man Conjuror Trendle (based on a real Dorset character) to have her afflicted arm stroked at the next hanging. ‘I used to send dozens for skin complaints’, he noted. ‘But that was in former times. The last I sent was in '13—nearly twenty years ago.’^6^ All but one of the ten eighteenth-century newspaper reports concerned London executions. This is hardly surprising considering the limited number of provincial newspapers in the period and the fascination with the spectacle of Tyburn and Newgate executions. But with the expansion of the regional press and local reportage during the nineteenth century we find cases from across the southern half of the country. There are three instances in Sussex, two each in Surrey and Warwickshire, and single instances from Kent, Wiltshire, Somerset, Gloucestershire, Staffordshire and Lincolnshire. References to the practice in other sources noted in this article extend the geographical range to Suffolk, Worcester and Northamptonshire.^7^ So in the ensuing discussion we have to bear in mind that we appear to be dealing with a regional tradition, whereas stroking with non-criminal dead hands was practised across the country.

We could consider this tradition, as others have done, as a curious example of folk medicine or an intriguing footnote in the study of execution ritual.^8^ In his study of the Tyburn riot against the surgeons, Peter Linebaugh related several examples of ‘gallows superstitions’, noting that their full significance could not ‘be assessed without a greater knowledge than we now possess’. Vic Gattrell similarly referred to the ‘real puzzles in scaffold ritual’. Andrea McKenzie's *Tyburn's Martyrs* has taken new steps with regard to the secular, symbolic and religious rituals that infused the execution scene, but there has not yet been an attempt to unlock the puzzle of scaffold ‘superstition’.^9^ As with other manifestations of popular medical and magical practice mentioned in passing as curiosities, mental relics or superstitions in the historiography, a closer reading reveals their complexity, and the multiple insights that can be drawn from them.

The hanged man's hand (and it is always a male appendage) beckons the historian to explore a range of issues regarding the changing relationship between popular and orthodox medicine, the meaning of the criminal corpse, and the rituals of healing and punishment from the seventeenth to the nineteenth centuries. The gallows touch was performed and reported upon in a period that, historians have argued, saw fundamental, connected, social transformations. These concern such themes as the separation of elite and popular cultures, the redefinition of public and private discourse, the diminishing influence of the established church, and the redefinition of the relationship between punishment and the body politic: in short transformations that define modernisation and enlightenment in British society.

As already noted, our understanding of the gallows touch is dependent on the rise of the newspaper in the eighteenth century, and its role as a vehicle for expressing enlightened opinion in a national public forum. The letters, reports and commentaries in the burgeoning national and provincial press chimed with the contemporary campaign to ‘civilise’ and reform popular cultures. This manifested itself most obviously in successful campaigns to suppress blood sports through legislation, and the efforts to tame and repackage popular celebrations.^10^ The antiquarians who related instances of the dead man's hand were inspired to record the beliefs and practices of the ‘common people’ as a marker of how far polite society had come from the days of ‘Catholic superstition’. The ‘vulgar’ traditions of the unlearned were worthy of record as mental relics, curios of a past world that would, with a tinge of regret, be lost as the light of reason reached the remotest corners of the land.^11^ This civilising campaign also dovetailed with the agendas of those who called for the abolition of public execution. While some saw the spectacle of public punishment as a valuable instructive tool, reinforcing collective social and godly justice, there were those who saw it as promoting the base, barbarous impulses that inhibited the development of a godly, humane society. Both sides would have agreed on the ‘disgraceful’ nature of the hanged man's touch, though.^12^

This article highlights how a popular healing tradition can inform and question our broader understanding of these historic developments and historiographical debates. It examines how and why the tradition continued in the closely controlled and politicised arena of the gallows. Indeed, the hanged man's hand helps justify the concept of the long eighteenth century and questions received definitions of the early modern and modern era with respect to criminal justice and medicine. This article is also an exercise in recovering popular medical traditions from the condescension of history. It explores the challenges and ‘known unknowns’ when attempting to decode ‘popular’ cures from the fragmentary evidence and the distorting lenses of the sources. This is, by its very nature, a speculative exercise, recognising the multiplicity of conceptions and interpretations that could have existed at an individual, family and community level in regional and religious contexts.

## Miraculous and Medical

Across early-modern Europe the corpse was a significant element in the pharmacopeia of the medical profession and the populace. Blood, bones, fat and sweat could be ingested, prepared and smeared in order to cure a variety of ailments from epilepsy to ulcers and rheumatic pains. Often based on classical medical theory, human organic substances were thought to possess physical and spiritual virtues that no animal, mineral or plant could provide.^13^ The touch of a dead hand was not based on the ingestion or absorption of corpse constituents, though, but on the efficacy of stroking. In one sense this had parallels with the miraculous divine touch. In Catholic contexts the power of the healing relic is obvious. This usually concerned the body parts or supposed body parts of saints and martyrs, but other traditions existed. In the Italian region of Abruzzo, for example, the hand of a recently deceased priest, preferably still warm, was thought to cure skin tumours.^14^ Protestant England had its own clerical healing hand—that belonging to Father Edmund Arrowsmith, a Jesuit executed at Lancaster in 1628. His hand was cut from the body and consequently became a ‘holy hand’, capable of effecting healing miracles upon those who came into contact with it. In 1737 a pamphlet related the recent cure of a 12-year-old boy named Thomas Hawarden of Appleton. The boy had suffered from smallpox, and had become paralysed and afflicted with impaired sight. Arrowsmith's hand was brought to his home and a miraculous cure effected by ‘stroaking it down each side of the Back-Bone, and then a cross’. The miracle was apparently attested by Protestants as well as Catholics. Further cures were recorded well into the nineteenth century.^15^

The divine laying on of living hands was expressed in the English and French royal touch for the King's Evil. The monarch's supposed ability to heal demonstrated to his or her subjects the divine right of dynastic monarchies. While practised in the medieval period, the custom sat awkwardly with Protestant theology so Elizabeth I rejected the tradition. It was briefly resurrected by Charles I, and came into royal vogue again for several decades after the Restoration.^16^ There were other humble men and women, of course, who also claimed the curative touch through divine inspiration or birth right, such as being the seventh son of a seventh son.^17^

Seventeenth-century physicians worked within a medical framework based on classical theory and empiricism that had not yet been fully disproven or disentangled from the concepts that lay behind common medical beliefs and practices. The power of the waxing and waning moon on the body, for instance, was generally accepted. So the Somerset physician John Allen (*c*.1660–1741) noted in 1730 that scrofula medicines worked best in the last quarter of the moon, its waning reducing the swelling. The waxing moon would have a reverse influence, and so treatment should be discontinued with the arrival of a new lunar cycle. A century later and the medical profession readily dismissed such astrological sympathies as mere superstition.^18^

Seventeenth-century medical explanations for the curative action of dead bodies followed two main competing frameworks—Galenism and Paracelsianism.^19^ Based on Paracelsus's experiments with corpses, English Paracelsians believed certain body parts had inherent chemical and spiritual curative qualities that could be transferred from fresh corpse to living person. The views of those who subscribed to Galenic medical theory and practice were more diverse, ranging from those who completely dismissed the notion, to those who accepted that some agency occurred but through the cadaver acting on the humours of the living. In a medical world subscribing to Galenic humoral theory it is understandable that the temperature of the dead hand rather than its essence was considered the active principle. This was evidently the view of William Harvey (1578–1657). He explained to his friend Robert Boyle that he ‘sometimes tried fruitlessly, but often with good success’, to cure tumours and excrescences with a dead hand. He was particularly successful when he kept the touch going for a ‘pretty while’ so that ‘the cold might thoroughly penetrate’.^20^

A few physicians were interested in uncovering the natural, scientific secrets of popular remedies that were generally held to work. So it was curiosity about cures ‘often approved by the common people’ that led the maverick occult philosopher and physician Robert Fludd (1574–1637) to consider the dead man's hand. ‘A dead bodies hand touching warts, they will dye’, he observed, likening it to the common cure for warts whereby they were rubbed with a piece of meat which was then buried.^21^ This was an act of sympathetic medicine, whereby two things which had been in contact with each other maintained a long-lasting imperceptible relationship. There was no potency in the hand then; like the meat it was merely a vehicle for transferring the affliction from a living organism to similar but decaying material—or as Fludd, an arch-critic of Galenic medicine put it, ‘things are sympathetically maintained in their being, that is to say, in their increase or vegetation’. Still, Fludd also apparently experimented with palingenesis, the notion that the macrocosm was imprinted in the microcosm of the body's constituents. It was reported that Fludd had calcined the skull of an executed criminal, dissolved the ashes in water and seen therein an image of the hanged man: dead material possessed the imprint of life.^22^

The arrival on English shores of the famed Irish faith healer Valentine Greatrakes (1628–83) in 1666, at a time when Charles II was promoting his own divine healing, instigated a considerable debate as to the healing properties of the living hand that also touched upon that of the cadaverous hand. There were several exchanges on the matter in the pages of the *Philosophical Transactions*, the journal of the recently founded Royal Society. A key scientific explanation for the efficacy of Greatrakes' stroking centred on the physical influence of friction on the effluvia, or flow of invisible material particles, of his patients.^23^ But could a dead hand emit beneficial rather than noxious effluvia? Psychological explanations were also put forward. John Quincy (*d*. 1722), a Dissenter and apothecary who dismissed the ‘superstition and bigotry’ of the royal touch, suggested nevertheless that there might be real curative value in the dead man's hand because ‘the Imagination in the Patient contributes much to such Efficacies, and because the Sensation which stroaking in that manner gives, is somewhat surprizing, and occasions a shuddering Chillness upon the part touched; which may in many cases put the Fibres into such Contractions, as to loosen, shake off, and dislodge the obstructed Matter’.^24^ That said, Quincy was clear that such a cure should not be practised by the profession. Similar opinions were expressed in lectures regarding the best cures for scrofula delivered in 1765–66 by the Scottish professor of medicine William Cullen (1710–90). While calcinated vegetables and burnt sponge were amongst his preferred applications, once again the weight of testimony led him to conclude that ‘fear and awe’ could have a beneficial physiological effect on the condition, as applied when ‘rubbing a dead man's hand, and the royal touch, which make a deep impression on children, from their solemnity’.^25^

This learned discourse and debate on the power of the dead hand was digested and regurgitated in a letter to the *London Chronicle* in 1759, published in response to a newspaper report of a stroking at a public execution the previous week. A young woman with a wen on her neck was held up to the gallows and the executed thief's hand was rubbed over the protuberance several times. The *London Evening Post* dismissed it as the ‘dregs of superstition’, but the *Chronicle*'s correspondent observed: ‘That the dregs of superstition do still remain among us, I have not the least doubt at all; nay, we frequently meet with the remains of Heathenish superstition. But that this action arises from superstition, I deny; and that because it is founded on philosophic principles.’^26^ He or she then went on to explain that the corpse had its own amount of ‘action’, which coincided with the beginning of the decaying process. The life inside the individual was preserved by the circulation of the vital juices: when death arrived the process was not stopped, but inverted, and the juices moved towards the external margins of the corporeal structure, producing a gradual putrefaction, that is the fermentation and dissolution of the matter. This process took some time before occurring and reaching the boundary of the body: then it could be transmitted through repeated or prolonged contact, and so dissolve the swellings on other diseased bodies. To validate this explanation the writer referred to Robert Boyle's reports. Thus, the author concluded, the fresh hanged man's hand could not actually heal, because death was too recent. The preferred moment for its performance and its location at execution places were therefore ineffectual.

Despite attempts to make scientific the long-held popular practice of the curative dead hand, there were explicit magical components in its popular application. The number of times the swelling was stroked was crucial—usually three, seven or nine times, numbers we find over and over again in magical healing rituals.^27^ Applying nine strokes seems to have been most common. As we saw in the introductory quote to this article, the hangman at the execution of the White brothers in 1758 abided by this numerical tradition. The eighteenth-century antiquarian Francis Grose also noted the practice of rubbing the afflicted part nine times with the dead hand. In the mid-nineteenth century it was reported that a Mrs Charles Standon, who had suffered for some years from a swelling on her throat, went to Waltham Lock, Hertfordshire, to be stroked by the hand of a drowned boy, ‘nine times from east to west, and the same number of times from west to east’. The folklorist William Henderson related an account from County Durham, where a woman, who had been suffering from a wen for eleven years, was advised by ‘a very respectable man’ to rub a dead child's hand nine times across the excrescence.^28^ Another healing tradition that applied to charmers and charming more generally, and which was also sometimes observed with the dead hand, was that the patient had to be a different gender to the healer). The seventeenth-century antiquarian John Aubrey noted this contra-sexual requirement, relating the story of how a Somerset painter who had a wen the size of a pullet's egg in his cheek was cured by rubbing it with a dead woman's hand.^29^ Similarly a Shropshire folklorist noted that a woman suffering from the King's Evil had to eat a piece of bread and butter from the hand of a killed man, and vice versa.^30^ It is worthy of note that there is no strong bias towards left or right hands in these traditions.

## Untimely Dead and Executed Criminals

So far in this discussion no distinction has been made between the curative power of the natural dead and the untimely dead. All corpses were thought to have the potential to have potency it would seem, as illustrated by the examples mentioned above and the judicial corpse of the murdered. In the latter tradition, which ended as a quasi-official, divine ordeal by the early eighteenth century, corpses of murdered people were believed to bleed if the culprit approached and touched them. The practice continued in less formal contexts into the nineteenth century.^31^ So, was the gallows corpse merely an accessible corpse? Or did the act of execution impart it with extra or different potency? Seventeenth- and eighteenth-century medical literature on dead man's hand therapy made little reference to the resort to the freshly hanged. Robert Fludd recalled having ‘a certain body of one that was hanged’ in his house to conduct a private anatomy. At the time, the College of Physicians, to which Fludd was admitted in 1609 after numerous failed attempts, was allowed to anatomise six criminal corpses every year for public anatomy. An acquaintance, an apothecary named Mr Kellet, heard of the corpse in Fludd's house and requested that Fludd allow a gentlewoman with a tumour in her belly ‘to be touched and stroked with the dead man's hand, because experience had taught it to be very efficacious, for the abolishing of the like horrid protuberation in others’. Fludd duly agreed and sometime later the woman's husband paid a call to Fludd to thank him and give the good news that the stroke had done his wife good.^32^ There is nothing in Fludd's account and explanations, though, that suggest he or those concerned thought the *criminal* corpse had more potency than a normal corpse. Yet this concept was undoubtedly known in early-modern medical circles, for it was noted in that much-cited classical source, Pliny the Elder's *Natural History*: ‘We are assured that the hand of a person carried off by premature death cures by a touch scrofulous sores, diseased parotid glands, and throat affections; some however say that the back of any dead person's left hand will do this if the patient is of the same sex.’^33^ It is noteworthy that Pliny's observation about the gender of patient and corpse is contrary to the English contra-sexual tradition mentioned earlier.

The executed were not the only ‘premature dead’ of course. There were the innocent type—those murdered, or those killed in battle or by accident. But in the southern half of the country it was clearly the criminal untimely dead that were most prized during the eighteenth and nineteenth centuries. This includes suicides or self-murderers. In 1853, for instance, a woman with a wen on her neck was advised by a wise woman to travel to Hesleden Dene, not far from Hartlepool, where a man had committed suicide by hanging himself. The corpse was laid in an out-house awaiting the coroner's inquest, and the afflicted woman was allowed to stay there all night with the chilled hand of the suicide on her wen.^34^ In July 1879 the *Western Morning News* reported that two elderly women, and a young boy suffering from the King's Evil, had recently attended a coroner's inquest on a suicide in Plymouth and asked for permission to use the hand of the suicide on the boy so as to effect a ‘perfect cure’. One of the women explained that a person she knew had her health restored this way, ‘but of course she could not say whether it was the hand of Almighty God, or not’. Other than suicide, though, there does not appear to be any link between the type of criminal act and the curative potency of the hand that committed it.

What was it about the criminal body that gave it extra potency to heal and protect in popular belief? If it was not a mere matter of humoral balances between cold and heat emanating from the afflicted and the corpse, or the physiological actions of friction or fear, what did those who went to be cured think was happening during the stroking at the gallows? One possibility concerns the medical-magic theory of transference. Maybe the criminal corpse was the most morally appropriate human vessel to which to pass on the affliction. What could be more efficacious than to transfer illnesses to those destined for the afterlife? In this sense it was the reverse of the tradition of sin eating, when an individual symbolically ingested the sins of those recently deceased by ritually eating bread and drinking over the corpse. Consider an account from the coast of Donegal, in Ireland, where the transference of disease merged with the Catholic theory of purgatory, the intermediate state that the soul had to cross painfully in order to atone for its sins. During a funeral a man suffering from rheumatism, applied the dead hand to his arm, shoulder and leg asking the dead to take all his pains with him, ‘in the name of God’. The sickness of the man would have been a small addition to the sufferings that the spirit of the deceased person had to endure in purgatory.^35^ Clearly, in this belief spiritual and bodily dimensions overlapped: the sinful soul of the dead easily attracted the physical impairment of the patient, while its final redemptive destination in Heaven assured the restoration of health.

In a general Christian context the action of the criminal corpse could also be considered an act of atonement as the last moments of life drained away and the soul left the body; the sinner performed an act of unwitting, deity-ordained goodness as he or she departed the mundane world. In this sense the hanged man's stroke was a mutual act of benevolence, an exchange of physical and spiritual succour. It is clear from comments already related in this article that at least some people viewed the stroking in religious terms, as a branch of the general tradition of the divine touch or divine intervention. It was all in God's hands. This would have been confirmed and endorsed by the scaffold sermons and confessions that emphasised ‘dying well’ through expressions of penitence either as ‘religious theatre’, as described by Sharpe with regard to the seventeenth century, or through expressions of personal moral culpability and regret. As Andrea McKenzie has explored, on the gallows the condemned expressed not only their personal guilt or innocence, but also, either vocally or symbolically, larger issues of societal guilt, sinfulness, redemption and social justice.^36^

Then again, it is likely that some did not rationalise the practice within a religious framework—or in terms of transference or decay. Maybe it was mostly about unexpressed secular notions of life forces transmitted through the blood and permeated through the skin. When, in Hardy's ‘Withered Arm’, Gertrude asks Conjuror Trendle how being stroked by an executed man could do her good, he replies, ‘it will turn the blood and change the constitution’. This was the language of the practice of blood-letting, which was still in vogue during the early nineteenth century, and also that of animal magnetism. One French magnetist reported that ‘when there is merely a local inflammation … it is easy to turn the blood from the direction it has taken: by drawing the fluid towards the legs and the feet’.^37^ The popular absorption of the basic theory of animal magnetism, touted through the late eighteenth and early nineteenth centuries, and used to explain the healing power of Greatrakes, may also have helped maintain the medical legitimacy of the hanged man's hand amongst some of the literate public. In this respect it is worth noting that several of those seeking the cure were described as elegantly and decently dressed or of ‘genteel appearance’. As various popular encyclopaedias and journals of the early nineteenth century related, animal magnetism worked through stroking, thereby transferring the invisible magnetic fluid of the magnetiser to the patient. Although not discussed in relation to the hanged man's hand, mesmerists gave some thought to the moment of death, mostly in relation to the departing magnetic life force acting upon the perceptive faculties of others, even over great distances. This manifested itself in loved ones seeing visions or sensing the death of the dying.^38^ Animal magnetist Jules Dupotet reported the view that ‘as long as the mass of the blood is warm, and not congealed, all the members of the body continue pliant, and as long as this is the case the soul remains in it’; the person is in a perfect trance; only when the brain and nerves lost their warmth did the soul separate itself.^39^ Death was not instant; for some minutes the hand of the executed remained connected to the soul. So from a mesmeric perspective the freshly executed criminal was still a magnetic being, the emotional, violent act of murder and then the anticipation of execution briefly boosting the life force of the criminal—rather as the Paracelsians had argued two and a half centuries earlier. This could be exploited by the living in a brief moment after execution.

Why the hand? Why not the stroke of a hanged man's foot? Clearly the basis of the cure was not just about the criminal body as a whole but also about the agency of the hand. Writing on the anthropology of the human hand in 1888, Frank Baker related some British cases of the dead hand stroke, and observed: ‘the hand is so intimately connected with the brain as the executor of its interests that the savage mind naturally ascribes to it a separate and distinct force independent of the rest of the body—makes it, in fact, a fetish’.^40^ It was not just the savage mind, of course. In the Aristotelian philosophical tradition the hand, the ‘instrument of instruments’, was the bodily metaphor for human as well as divine action; it had a liminal quality as an object of action and as an expression of ‘interiority, intentions, and inventions—of the self’.^41^ As we have seen, the ‘healing hand of God’ was integral to Christian healing traditions either as a metaphor or manifest in the form of the divinely gifted stroker. With regard to the gallows corpse, the hand had a dual significance as the means of both the criminal act and the healing act; it held the knife, the gun, the stone that killed. It was the hand not the foot that enabled the forgers William Dodd and Lord Massey (both of whose corpses were used for stroking) to do their artful work, and hands were the principal tools of the burglar and horse-thief. In the condemned cell confession of the sheep-stealer Edward Clarke, hanged at Chelmsford in 1814, he requested that ‘three of my fingers be taken from my hands, to be given to my three children as a warning to them, as my fingers were cause of bringing myself to the gallows’. A surgeon duly complied after the dissection.^42^ The magical properties accruing to the hand because of its criminal agency lay behind the tradition of the Hand of Glory. This was the hand from the corpse of a hanged criminal, which was drained of blood, dried and preserved. When it held a lit candle made of the fat of a hanged criminal it stupefied those in its presence; its principal purpose being to commit robberies.^43^ Here the post-mortem criminal hand used to commit further crimes was potent when detached from the corpse and desiccated, whereas the power of the hanged man's hand as benign, healing agent lay in its attachment to the body still replete with blood.

Why a male hand? The answer may be purely a matter of statistics. We cannot discount the basic fact that the vast majority of the hanged were men (around 94 per cent [6,069] in England and Wales between 1735 and 1799).^44^ Still, female hangings were not a rare sight in London in the period concerned. Perhaps the absence of the female touch was a consequence of broader societal sensitivities regarding the post-mortem treatment of the executed female body, the same sensitivities that also dictated the reluctance to gibbet women.^45^ It is likely that the contra-sexual healing tradition also had a significant influence. In all 27 cases mentioned at the beginning of this article the executed were men but only two of the patients were adult males. The gender bias in terms of those seeking the cure might also have been defined by the greater female concern over physical blemishes and the scarring caused by wens and other swellings.

## Location, Spectacle and Process

Transference, life force and, to a lesser extent perhaps, the spiritual arguments, all explain why the freshly executed were sought out and not gibbeted criminals. The gibbet corpse was already in an obvious state of decay, thus rendering transference less potent. Its life force was well and truly extinguished by the time it was creaking in its cage. The soul had long left the body. But the act of execution is also central to the complex of beliefs. As Gattrell observed, the gallows was a place where miracles might occur and ideas of damnation mingled with hopes for redemption.^46^ The hanged man's hand was not only potent because of the innate vitality and materiality of the corpse or the fate of the soul; the location and apparatus of execution also imbued it with potency and *vice versa*. Splinters from the gallows were thought to have protective and healing qualities. Writing in 1650 Sir Thomas Browne noted that to cure the ague ‘we use the chips of the gallows and places of execution’. The hanging rope was thought to cure headaches, as the witchcraft sceptic Reginald Scot observed back in 1584, and it was also thought to promote good luck. John Aubrey and Grose also noted its supposed power. The Georgian antiquarian John Brand (1744–1806) recalled seeing a hanging at Newcastle when just after the body had been cut down several men scrambled up the gallows to get the rope. After the Leeds poisoner and satanic-pact maker William Dove was executed in York in 1856, the hanging rope was carefully placed in Dove's coffin to ensure it was not cut up and sold off.^47^ While in some instances this was a matter of celebrity trophy hunting, the desire for gallows magic also drove demand.

There was a purely pragmatic reason for resorting to the gallows for the dead hand stroke: it provided the best opportunity for pre-meditated access to a very fresh corpse. The sufferer could plan ahead. The time and location of the assizes, and details of those being tried for capital offences, were known days or weeks in advance via the press and word of mouth. This was described well in Hardy's ‘Withered Arm’ with Gertrude seeking out news as to the next assizes. When she heard they had been held ‘she inquired stealthily at the inn as to the result’, then realized that there was not enough time for her to make arrangements to attend the ensuing execution. Under the Murder Act of 1752 all convicted murderers had to be hanged within forty-eight hours of sentencing, unless the sentence was passed on a Friday or Saturday (hangings being forbidden on Sundays). By comparison, getting to a suicide's corpse within minutes of death was highly unlikely, and no doubt many families would have been reluctant to have strangers traipsing to their door requesting to be stroked by their beloved ones. Still, the same sentiments were also expressed by some of the families of the executed, though to a lesser extent. So, in 1819, at the execution of a Jew named Abraham Abrahams on Penenden Heath in Kent, an application to be stroked was refused by the other Jews attending the corpse, because ‘they could not suffer the body to be touched by any but their own people’, ‘it being contrary to their customs’. At the hanging of John Highfield at Stafford in August 1828, some women, who had travelled a long distance to apply the dead hand to their necks, were violently opposed by the executed man's daughter.^48^

Yet the ritual and spectacle of the execution enhanced the perceived potency of the healing transaction, with the Murder Act of 1752 opening up a new public relationship with the criminal body. Through expanding the application of gibbeting or hanging in chains, the swinging corpse became a more permanent and widespread fixture in the public consciousness over the next 70 years. Furthermore, the much more common post-mortem punishment by public dissection reinforced the notion of the cadaver as a medical resource. The Act also led to a significant increase in hanging days in London.^49^ All in their way promoted greater public interaction with the hanged, and may have normalised a consensual post-execution relationship with and use of the criminal corpse. This would help explain why the resort to the hanged man's hand seems to have become more frequent from the 1750s onwards.

The corpse and the patient were the principal actors but the staging and the audience also worked their magic. The repeated spectacle of the gallows stroke can be seen as an example of what Thomas Laqueur has described as the ‘dramatic inconsistences’ of the English hanging, which demonstrate that it was not the state but ‘the people’ who were at the heart of the public execution in a ‘carnivalesque moment’.^50^ It is important to consider how the stroking was engineered in this respect. It was no mere matter of a quick dash to the scaffold and a grab at the dangling hand. The approach was usually made around fifteen minutes after the drop, time being given to ensure the victim was dead—there was no curative value in a half-hanged criminal's hand. The reach for the hand could present a challenge. During the mid-eighteenth century hangings were still taking place from tree boughs or beams reached by a ladder, or with the criminal standing on a cart which was then driven away, leaving the body to hang. The height of the swinging corpse sometimes meant it was difficult to reach from the ground, so at a hanging in 1759 a woman seeking the touch was held up in a man's arms, likewise in May 1767 after the execution of the murderer Francis Gorman a young woman had to be lifted up to have the dead man's hand rubbed upon a large wen on her neck.^51^ One magazine correspondent recalled attending a hanging at Worcester sometime in the mid-eighteenth century and ‘saw many females raised up on the shoulders of men, to have wens on their necks stroked’. It was not a very dignified procedure, and the same correspondent noted ‘the occasion was sadly deteriorated, by the ludicrous faces of some of the young girls, in their struggling efforts’.^52^ The adoption of the ‘New Drop’ technique introduced at Newgate in 1783, with the top half of the hanging body accessible above the trap door made performing the stroke easier. Change was slow, though. When two young women were stroked by the hand of the burglar Robert Bignall, at Horsham in 1807, the authorities were still using the cart method—Bignall requesting of the hangman in a low voice that he hoped he ‘would not make a bungling job of it’.^53^

As described by Simon Devereaux, towards the end of the eighteenth century the authorities increasingly limited the interplay and propinquity between the crowd and the act of hanging while reinvigorating the ‘theatrics of execution’.^54^ Scaffolds were built higher, such as the ‘false stage’ introduced at Newgate, or moved to the rooftops as at Horsemonger Lane prison in Surrey. This had the effect of formalising the hanged man's stroke, rendering it more ritualised, more of a public spectacle, and as a consequence imbuing it with more decorum and official legitimacy than previously. The patient had to obtain access to the stage, walk across it in full gaze of thousands looking up, and with secular and religious officials looking on, and go through the ritual. There is no sense from the accounts that the crowd reacted strongly to this secondary performance after the main act. A shocked newspaper correspondent of the *Leeds Mercury*, attending a London hanging in 1825, looked around him when witnessing a stroking ritual and found that ‘the mob did not appear to participate in my astonishment at beholding this sight, from which I infer that it is by no means uncommon. Now what do you think of this?’^55^ The fear and distress of the women being stroked was remarked upon several times, though. In 1808, for instance, the *Morning Post* reported regarding a lady of ‘genteel appearance’ who went to be stroked by the hand of the robber Abraham Brace at the Horsemonger Lane scaffold, ‘we never saw horror more feelingly depictured, in real life, than in her countenance’. The journalist continued, ‘If, as has been said, extreme terror in the party so acted upon, will give the recipe some effect, this lady will have a good chance of relief’.^56^

While the corpse and the patient were the dramatic leads, the hangman was best supporting actor, though some performances were very poor indeed. At Gloucester in April 1837, several women were allowed on the platform after the hanging of the murderer Charles Samuel Bartlett to receive the dead hand stroke. The moment was turned into a grotesque performance by the drunken behaviour of the hangman, who repeatedly mocked the corpse, shook hands with it, boxed him on the ears to make him swing around, and removed the cap from his face.^57^ This spectacle was widely reported in the national and regional press, was referred to in the House of Commons, and used by abolitionists to illustrate the barbarity of public executions. Locally some doubt was cast on the veracity of the report, and the High Sheriff launched an investigation and took depositions.^58^

As is already evident, the whole healing process could not have taken place without the hangman's aid and authority. He was the mediator between two rituals, the judicial and the curative, one representing the authority of the state, the other the cultural rights of the common individual. In this role he was an active member of the cast; he was not merely there to sanction access to the corpse, he also facilitated the healing act. At the execution of the murderer Patrick Welch at Newgate in 1825, an old woman was allowed to step onto the scaffold helped by a younger person. The executioner then ‘placed his arm round her neck, and proceeded to rub it with the hand of the malefactor; he continued to do this until the poor old lady had nearly fainted away, then he desisted, but, after the lapse of a short time, renewed his exertions with the other hand.’ At the execution of John Holloway at Lewes in 1831 William Calcraft let a man ascend to the scaffold, sat him on the edge, loosened Holloway's hands and rubbed the palms across a wen on the man's forehead.^59^

In many European countries the executioner's trade was considered dishonourable, and those recruited to the role were often former criminals; hence they shared with the executed not only the main scene at the scaffold, but also a similar moral background. A central figure in the theatre of execution, he was marginal and marginalised in other social contexts because of his polluted status.^60^ In Spain, Sardinia and in Southern Italy the hangman could not touch anything displayed in the markets and used a stick to point at the things he wanted to buy.^61^

Although executioners were only the means through which society enacted capital punishment and not the actual murderers of the convicted, their physical proximity to the corpse, and their treatment of it, placed them in an ambiguous zone between existence and the evidence of mortality, between human justice and the terror of the spectacle of death, between the fate of the matter and that of the soul. They worked at a boundary where life entered death, and where an individual surrendered to spiritual liberation. In this respect the executioner has been compared with the royal ruler: at the moment of execution he ‘stood as the sovereign's representative, an embodiment of sovereign power’.^62^ One was a common object of disgust and fear, the other of honour and respect, but both were possessed of sacral aspects and thaumaturgic powers. The royal potency was divine whereas as the executioner's was accrued through association and action. So in some parts of Europe (not England) the executioner's touch itself was seen as a substitute for his victim's, while in Liège, to touch the clothes of the hangman was thought to cure cysts.^63^

In England, the hangman's medicinal reputation was minimal compared to his continental brothers, in part due to the nature of inquisitorial Roman law and different execution and post-mortem practices that allowed body parts and blood to be appropriated. In early-modern German states, for instance, it was usual for the executioner to double up as torturer, and consequently the executioner was thought to have an intimate knowledge of human anatomy and the limits of bodily endurance. The executioner was also charged with nursing the tortured to ensure they survived to undergo trial. As a result, they were thought to be excellent bone-setters, and were officially recognised as such in mid-eighteenth-century Prussia. In the Netherlands, cases of hangmen practising medicine are found up until the late eighteenth century. German executioners also practised dissection which further enhanced their reputation as students of the human body, and provided them with legitimate sources of body parts for natural as well as magical healing. As late as 1747 one executioner petitioned to continue his dissection trade because it was the best source of human fat, with which he had cured numerous people in his town. The close relationship between healing and executioner was such that healers sometimes sought or were sought out to become executioners.^64^

English hangmen, by contrast, were primarily vendors and facilitators rather than healers *per se*. We know that they made money from selling ropes for healing and magical protection. John Aubrey amongst others had noted this trade in the seventeenth century. The *Spectator* observed with disgust that the hangman of William Corder, the murderer in the sensational Red Barn Murder case of 1827, had auctioned sections of the rope. The scene was depicted by the celebrated cartoonist Cruickshank, who has the executioner asking for a guinea an inch, while a member of the crowd states, ‘I want some of it for the university’.^65^ Money changed hands for facilitating the stroke as well. The early nineteenth-century hangman James Botting, was, for a time, allowed to apply the hanged man's hand to people with skin complaints for a fee of two shillings and six pence. In 1831 William Calcraft was still charging the same, which was also the fee he charged for whipping a criminal.^66^ Cost was evidently built into the decision making of those seeking the cure. The Northamptonshire folklorist Sternberg provides a rare insight when he observed that at one of the last public hangings in Northampton the applicants for the ‘dead-stroke’ were very few ‘not so much in consequence of decrease in faith, as from the higher fee demanded by the hangman’.^67^

## End of Spectacle and the End of a Tradition

In 1828 a newspaper article praised reforms that had taken place at Newgate which had the effect of ‘banishing a most disgusting indecency’—the hanged man's stroke. It related that the former governor Joseph Brown, who appears to have taken up the post in 1817, forbade any but officials to approach the suspended corpse and also ended the practice of allowing the hangman to keep the clothes of the deceased.^68^ But the situation was more complex than this suggested.^69^ In June 1818 the recently appointed Newgate executioner James Botting, having just hanged John Davey and George Claxton, chased after the Sheriff's retinue to complain that Brown had tried to deprive him of one of his perquisites—in other words admitting women to be rubbed with the hand. Offended by the loss of income, he told the Sheriff, Sir George Alderson, that he considered him and not Brown his master, and requested he be allowed to perform the service. Alderson asked if there were people currently waiting for it, and on being told there were two he informed the hangman that he could continue to perform the ‘unpleasant ceremony’.^70^ Brown evidently put his foot down subsequently, for we find Botting complaining to the Court of Aldermen in November that once again he had been barred from ‘the privilege of rubbing persons afflicted with wens’. The battle between the Newgate hangmen and their masters continued in 1824. At the execution in July of John Williams, John Reading and Thomas Davis for stealing and burglary, several people waited to be touched by the new hangman James Foxen, including a ‘lady of respectability’. The Sheriff, Sir Peter Laurie, explicitly ordered his officers not to allow the practice. It was a relic of the days of superstition and ignorance he said, ‘a custom which ought to be abandoned in this enlightened age; at all events he would do all in his power to do away with such a ridiculous practice.’^71^ Yet the following year Foxen assisted an old woman on to the scaffold and stroked her with the hand of the dangling Patrick Welch. This seems to be the last occasion in which the healing tradition took place on the London scaffold. It did not mean an immediate cessation of requests for Newgate healing, though. One of what must have been numerous requests during the 1830s was reported in July 1839 at the execution of the 18-year-old William Marchant who was convicted for murder. A woman asked to have her neck rubbed by the dead hand to remove a tumour, but if the cure was attempted it certainly did not occur in front of the public.^72^

It took a few years for the rest of the country to follow Newgate's example, but the pressure from the provincial press and its readers was building, just as the tide was turning on the morality and efficacy of public execution. In August 1824 the *Northampton Mercury* published a letter expressing disgust that this ‘species of superstition is tolerated and authorised throughout the country’. Provoked to write to the press by reports of the imminent execution at Northampton of Charles Clutton for sodomy, the author recalled attending the last execution in the town, presumably that of William Gent, William Meadows and Redmund Middleton for rape in 1822, where ‘a party of women and children afflicted with wens, ascended the platform to have them rubbed with the criminals’ hands'.^73^ When the stroking of two women and a baby took place at a triple hanging at Lincoln in 1830 the *Stamford Mercury* expressed regret ‘that Sheriffs do not give orders to prevent the display of so disgusting an imbecility’.^74^

In 1831 we find the famed William Calcraft, who became the Newgate hangman in 1829, taking liberties in the provinces at the execution of John Holloway in Lewes, Sussex. The Sussex Under-Sheriff warned Calcraft, however, that he would not ‘suffer a repetition of such proceedings until after the body was cut down’. It was reported that 23 thousand people filed through the magistrates' room in Lewes town hall to see the corpse, and no doubt a few requested the stroke.^75^ In 1837 women were allowed on the scaffold in Gloucester, and at the execution of James Crawley at Warwick in 1845 the ‘scaffold was crowded by members of the “fairer sex”’ being rubbed for various swellings.^76^ But during the 1830s we can detect a general shift in policy by the authorities that followed that of the Sussex Under-Sheriff; namely, the hangman could keep his perquisite but the healing act could only be performed away from the scaffold and the gaze of the crowd. So after the execution of James Cook at Leicester in 1832, the body was cut down and placed in the gaol for public viewing and those who queued to see the corpse were able to have their wens rubbed.^77^ What is unknown is whether in popular perception this lessened the potency and therefore desirability of the hanged man's hand due to the length of time since the execution, and the lack of spectacle.

The last permitted public stroking seems to have taken place at Warwick in April 1845, when several women were allowed to ascend the scaffold to have their wens rubbed, a local journalist describing the scene ‘as extraordinary as it was revolting to behold’.^78^ By the 1850s the authorities had put an end to the practice altogether with prison governors rather than Sheriffs taking the lead by tightening up access to the scaffold, and removing the executioners' discretion to allow access, just as governor Brown had attempted at Newgate three decades before. It is understandable that governors were more proactive. The Sheriff or Under-Sheriff was responsible for appointing and paying executioners, the expenses being claimed back annually from central government. The task of recruiting from a small pool of available ‘quality’ hangmen would have been eased by allowing them to continue claiming their much frowned upon but compensatory perquisites. The governor was in charge of security at hangings and of ensuring the execution was conducted humanely and efficiently: the performance of the stroking cure threatened all these responsibilities. Considering the growing national debate about public execution, and the shift of hangings to prison precincts, the governor's prerogatives were becoming increasingly important in relation to those of the provincial Sheriff. At the execution of William Wright at Reading gaol in 1855, presided over by Calcraft, a woman tried to force her way to the scaffold but was stopped by the police.^79^ When John William Beale was condemned to be hanged by Calcraft at a scaffold erected at the gateway of Wilton gaol, Taunton, in January 1858, a man from Bath, who had a wen upon his neck, applied to receive the dead hand stroke, but his request was rejected by the governor of the prison, Mr Oakley. Nevertheless, according to the *Bristol Mercury*, the man showed up the morning of the execution only to be once more disappointed by the renewed refusal.^80^ In April 1863 an old woman from Tachbrook, Leamington Spa, and her daughter, who suffered a large unsightly wen on her throat, travelled the few miles to Warwick prison for the hanging of the murderer Henry Carter. They called at the Porter's Lodge to request the governor to allow the hanged man's hand to be passed over her throat. Permission was not granted. This seems to have been the last reported application for the cure.^81^ Public hanging was abolished in the United Kingdom five years later, putting an end once and for all to a healing tradition that was both extraordinary and mundane, that became institutionalised and yet reviled.

Considering the hanged man's hand as a ‘superstition’, even referring to it as a ‘superstition’, has been one of the barriers to historians exploring its significance and meaning. This detailed reflection on the gallows cure supports the point made by Davies, with regard to satanic criminal inspiration, about the entrenched nature of ‘early modern’ popular supernatural discourses and motifs in judicial language, thought, and procedure during the long eighteenth century.^82^ The web of Enlightenment narratives still catches historians studying the social and criminal history of the era. As we have seen, eighteenth- and early-nineteenth-century commentators could come away from a multiple hanging, perhaps poorly executed as numerous hangings were, and still find the hanged man's stroke more revolting, shocking and disgusting than the hanging itself. Today the historian does not express such emotional gut responses to this curative tradition, but it has still been placed beyond the realms of our present understanding, something that is far less explicable, say, than the conception of public execution as a collective cathartic experience. From a medical history perspective, the healing power of the hanged man's hand, like much of our medical knowledge, has an origin in antiquity and yet reaches into the modern era. While women stood on men's shoulders reaching for the dangling hand, medical men were still seeking scientific explanations for its efficacy through the theoretical understanding of their day. A simple dichotomy between popular and orthodox medical theory and practice eventually emerges through the period concerned, but it is not a clear narrative. The stroke of the criminal corpse touches on some sensitive and insensitive parts of our own experience of the past.

## Funders

This work was supported by the Wellcome Trust as part of the ‘Harnessing the Power of the Criminal Corpse’ project (grant number 09590412fi11Z).
